# Fatigue Assessment in Patients with Hereditary Hemochromatosis: First Use of the Popular Diagnostic Tools

**DOI:** 10.3390/jcm13185544

**Published:** 2024-09-19

**Authors:** Michał Świątczak, Alicja Raczak, Agata Świątczak, Krzysztof Młodziński, Katarzyna Sikorska, Anna Jaźwińska, Damian Kaufmann, Ludmiła Daniłowicz-Szymanowicz

**Affiliations:** 1 Department of Cardiology and Electrotherapy, Faculty of Medicine, Medical University of Gdańsk, Smoluchowskiego 17, 80-214 Gdańsk, Poland; michal_swiatczak@gumed.edu.pl (M.Ś.); krzysztofmlodzinski@gumed.edu.pl (K.M.); d.kaufmann@gumed.edu.pl (D.K.); 2 Clinical Psychology Department, Faculty of Health Sciences, Medical University of Gdańsk, 80-214 Gdańsk, Poland; 3 Department of Pediatrics, Hematology and Oncology, Faculty of Medicine, Medical University of Gdańsk, 80-214 Gdańsk, Poland; 4 Department of Tropical Medicine and Epidemiology, Faculty of Health Sciences, Medical University of Gdańsk, 80-214 Gdańsk, Poland; ksikorska@gumed.edu.pl; 5 Regional Blood Donation and Hemotherapy Center, 80-210 Gdańsk, Poland; ania@gumed.edu.pl

**Keywords:** hereditary hemochromatosis, iron overload, HFE gene, fatigue

## Abstract

**Background:** Hereditary hemochromatosis (HH) is a genetic condition with fatigue as an essential but not precisely assessed symptom. While some well-specified scales for fatigue assessment in some pathologies exist, data on their usefulness in HH need to be collected. This research aimed to evaluate fatigue in HH using the Fatigue Assessment Scale (FAS), Fatigue Severity Scale (FSS), and Chalder Fatigue Scale (CFQ). **Methodology:** Seventy-nine HH patients underwent a questionnaire containing items about detailed medical history and the FAS, FSS, and CFQ scales. Twenty-five sex- and age-matched healthy persons constituted the control group (controls); additionally, thirty blood donors (donors) were compared. **Results:** The fatigue indices were significantly worse in the HH patients than in the controls and donors (HH vs. controls *p*-value: FAS = 0.003, FSS < 0.001, and CFQ = 0.003; HH vs. donors *p*-value: FAS = 0.025, FSS < 0.001, and CFQ = 0.041). There were no differences between the severity of fatigue and the specific genotype or the age of the patients. The HH women presented more severe fatigue than the men. High internal consistency and reliability for each scale were revealed: the Cronbach alpha values were as follows: FAS 0.92, FSS 0.95, and CFQ 0.93. Additionally, the construct validity and factorial validity of the implemented scales were confirmed. **Conclusions:** The HH patients exhibited significantly worse fatigue across all the scales. The FAS, FSS, and CFQ are simple and reliable diagnostic tools for assessing and quantifying fatigue for clinical and research purposes.

## 1. Introduction

Fatigue is a non-specific symptom that is difficult to evaluate objectively and accompanies many diseases [1]. Therefore, several scales are used to assess both mental and physical fatigue in different periods of disease and the differentiation of fatigue from depressive disorders [2,3,4]. A well-performed fatigue severity assessment provides additional information regarding the patient’s clinical status [1]. The data available in the literature mainly concern depressive disorders, cancer, sarcoidosis, multiple sclerosis, post-stroke, and heart failure as conditions in which fatigue scales could be of great clinical value [5,6,7,8,9,10]. When performed regularly at the beginning, during, and at the end of the treatment, such scales can track the effectiveness of the treatment.

So far, many scales have been designed to analyze the degree of fatigue among patients with various ailments [5,6,7,8,9,10]. A good example is the Fatigue Assessment Scale (FAS), which is successfully used for assessing patients with sarcoidosis [7,11,12]. The FAS is a 10-item tool with high efficiency and reliability, as shown in many studies (Cronbach’s α = 0.9) [10,11,12]. Another popular scale is the Chalder Fatigue Scale (CFQ), an 11-item scale that enables the assessment of physical fatigue (measured by items 1–7) and mental fatigue (measured by items 8–11) [4]. The reliability of the CFQ is high, with Cronbach’s α ranging from 0.83 to 0.9 depending on the scoring method [13]. Another example is the Fatigue Severity Scale (FSS), which is characterized by high internal consistency and reliability, with an average Cronbach’s α coefficient of 0.93 [14]. The FSS is a nine-item scale that provides answers on a scale of 1–7.

In the context of iron disorders, fatigue is a crucial clinical symptom. The feeling of fatigue is strongly associated with iron deficiency, one of the most common nutritional deficiencies in the world. Patients have described it as a deterioration of the motivation to perform daily tasks, a feeling of physical tiredness, or problems with concentration [15,16]. Chronic fatigue can also accompany diseases related to iron overload; however, the pathophysiology of fatigue in these cases has not been well described [16]. The toxic effect of iron, leading to oxidative stress, appears to be an essential fatigue source due to the further damage of various organs and the promotion of neurodegenerative disorders by oxidative stress [17]. Additionally, excessive iron levels in the human body can gradually disrupt endocrine gland activities, resulting in diabetes, hypothyroidism, and hypogonadism, all of which may lead to fatigue [18,19,20,21,22]. Iron overload may also result in the development of heart failure, in which fatigue is a substantial symptom [15].

One of the most common iron overload pathologies is hereditary hemochromatosis (HH), a genetic disease caused in 80% of cases by a mutation in the HFE gene [16]. Before discovering the HFE gene, the main signs of HH were cirrhosis, diabetes, and a dark skin tone [16]. Nowadays, genetic testing is available in the early stages of the disease, and one of the first symptoms is fatigue. Therefore, its precise evaluation is essential from a clinical perspective. So far, there are no reports in the literature regarding the objectification of such an important early symptom of HH. Additionally, the fatigue assessment scales have yet to be implemented for this group of patients. This paper aims to assess the usefulness of the popular fatigue scales (Fatigue Assessment Scale, Fatigue Severity Scale, and Chalder Fatigue Scale) in creating a fatigue profile of HH patients, determining the degree of fatigue and evaluating the impact of various factors on the level of tiredness in these patients.

## 2. Materials and Methods

### 2.1. Study Population

Consecutive patients with HH (diagnosed based on clinical characteristics, abnormal iron turnover parameters, and the presence of HFE gene mutations [23]) underwent a questionnaire containing items about detailed medical histories, including mutation, laboratory results, and duration of HH and administered treatments with laboratory parameters, and containing three fatigue assessment scales: the Fatigue Assessment Scale, the Fatigue Severity Scale, and the Chalder Fatigue Scale. Forty-nine patients (62%) had the C282Y/C282Y mutation, sixteen (20%) had the C282Y/H63D mutation, twelve (15%) had the H63D/H63D mutation, and three (3%) had the C282Y/WT mutation. The exclusion criteria were age <18 years old, left ventricular ejection fraction < 50%, a history of any cardiac diagnosis (apart from hypertension), and features of heart damage. The control group comprised of twenty-five healthy age- and sex-matched volunteers. Additionally, those people were not regular blood donors. Due to cyclical blood donation resembling venesection therapy, a group of 30 blood donors was included in the study. The introduction of blood donors as a second control group aimed to address how regular blood donation, which resembles venesection therapy in intensity, affects the severity of fatigue in healthy individuals compared to those with an HFE gene mutation. Given the stringent health requirements of this group of people, their age was lower than that of HH patients and controls. The study protocol was approved by the Local Ethics Committee of the Medical University of Gdańsk (NBBN/452/2016), and informed consent was obtained from all study participants.

### 2.2. Questionaries

In our study, we selected FAS, FSS, and CFQ scales due to their widespread availability, high reliability, internal consistency, and ease of use, enabling quick yet accurate patient assessment.

1. The Fatigue Assessment Scale (FAS) is evaluated using a scoring system where each response can be rated from 1 to 5, corresponding to the following frequency: 1 = never, 2 = sometimes, 3 = regularly, 4 = often, and 5 = always. However, for questions 4 and 10, the scoring is reversed: 5 = never, 4 = sometimes, 3 = regularly, 2 = often, and 1 = always. The cumulative score for the FAS ranges from 10 to 50 points. Based on the total score, patients are categorized into two subgroups: fatigue (scores 22–34) and extreme fatigue (scores ≥ 35).
2. The Chalder Fatigue Scale (CFQ) can be evaluated using two distinct scoring methods. The first is the bimodal scoring method, where responses are scored as follows: less than usual—0, no more than usual—0, more than usual—1, and much more than usual—1. The second is the Likert scoring method, where responses are as follows: less than usual—0, no more than usual—1, more than usual—2, and much more than usual—3. The possible score range for the bimodal method is 0 to 11 points, while for the Likert method, the range is 0 to 33 points [11]. In both methods, a higher score indicates a greater level of fatigue.
3. The Fatigue Severity Scale (FSS) consists of 9 items, each rated on a scale from 1 to 7, where 1 represents “strongly disagree” and 7 means “strongly agree”. The total score is calculated by summing the points received across all items, with a higher total score indicating a greater level of fatigue.

The translation procedure of the FAS, CFQ, and FSS in the Polish language followed the standard translation-back-translation procedure (Figures S1–S3) [24]. The Polish version of FAS developed for this research was consistent with the version available on the website of the World Association for Sarcoidosis and Other Granulomatous Disorders. (https://www.wasog.org/education-research/questionnaires.html (accessed on 18 August 2024)).

### 2.3. Statistical Analyses

Continuous data are presented as the median (25th–75th percentiles), while categorical data are expressed as proportions. The Shapiro–Wilk test was performed to determine the normal distribution of our data. Most of the analyzed parameters did not have normal data distributions, even after logarithmic data transformation; thus, we selected appropriate statistical analysis methods based on non-parametric tests. Comparisons between all groups were performed by the Kruskal–Wallis test for continuous variables (with Dunn’s post hoc test for the multiple comparisons with Bonferroni adjusted). The psychometric tests of the questionnaires included reliability (internal consistency, Cronbach’s alpha), factorial validity (principal component analyses with varimax rotation), construct validity (Spearman’s correlations between the scales), and validity (differences between patients and the control group). *p*-values < 0.05 were considered significant. Statistical analysis was conducted using R 4.2.1. environment (R Core Team, Vienna, Austria).

## 3. Results

Seventy-nine consecutive patients diagnosed with HH between 2001 and 2023 were enrolled in the study. Forty-nine patients (62%) had the C282Y/C282Y mutation, sixteen (20%) had the C282Y/H63D mutation, twelve (15%) had the H63D/H63D mutation, and three (3%) had the C282Y/WT mutation. The median age was 44 years (34–55). Table 1 shows the demographic data, medical history, and laboratory results of the enrolled patients.

### 3.1. Analysis of Fatigue among Patients with Hereditary Hemochromatosis

**Figure 1:**
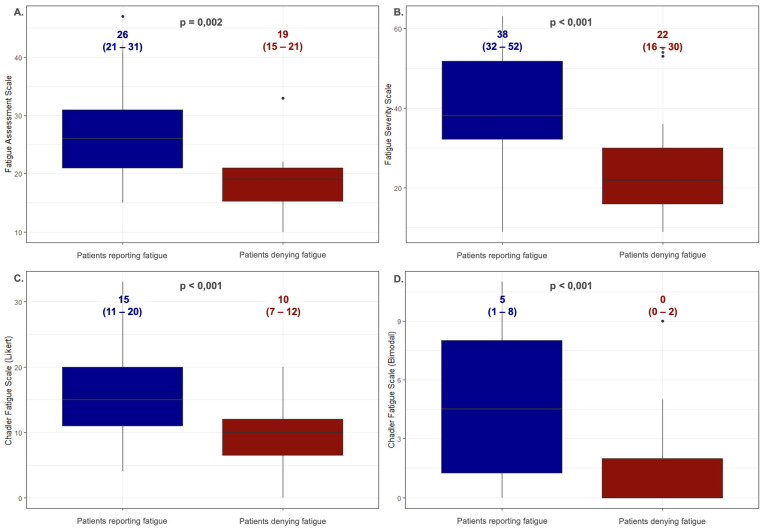
Comparison of the values obtained on fatigue scales by patients with HH divided by declared feeling of chronic fatigue (the U Mann–Whitney test). Data are presented as the medians (25th–75th percentile). (**A**)—Fatigue Assessment Scale; (**B**)—Fatigue Severity Scale; (**C**)—Chalder Fatigue Scale (Likert scoring method); (**D**)—Chalder Fatigue Scale (bimodal scoring method).

Figure 1 shows the values obtained on the fatigue scales by the patients with HH divided by a feeling of chronic fatigue (the U Mann–Whitney test). Fifty-five (71%) HH patients experienced chronic fatigue and obtained significantly higher scores compared to those patients who denied this symptom on each of the scales—for FAS: 26 (21–31) vs. 19 (15–21), *p* = 0.002; for FSS: 38 (32–52) vs. 22 (16–30), *p* < 0.001; for CFQ (Likert): 15 (11–20) vs. 10 (7–12), *p* < 0.001; for CFQ (bimodal): 5 (1–8) vs. 0 (0–2), *p* < 0.001. Sixty-four percent of the people reporting chronic fatigue were patients with the C282Y/C282Y genotype. In the second group, the C282Y/C282Y genotype was present in 50% of the patients. Additionally, no statistically significant differences were observed in the iron and ferritin levels between the groups (*p* = 0.788 and *p* = 0.461, respectively). There were no statistically significant differences in the age of the compared groups (45 (41–58) vs. 43 (32–55); *p* = 0.239).

The Spearman’s correlations between the FAS, FSS, CFQ, and clinical and laboratory parameters of the HH patients are provided in Table 2. A correlation between the ferritin levels and scores in FSS was observed. There was also a correlation in the results obtained from the CFQ with the year from diagnosis. The correlations were significant; however, they were relatively weak (the r-values were lower than 0.7).

**Figure 2:**
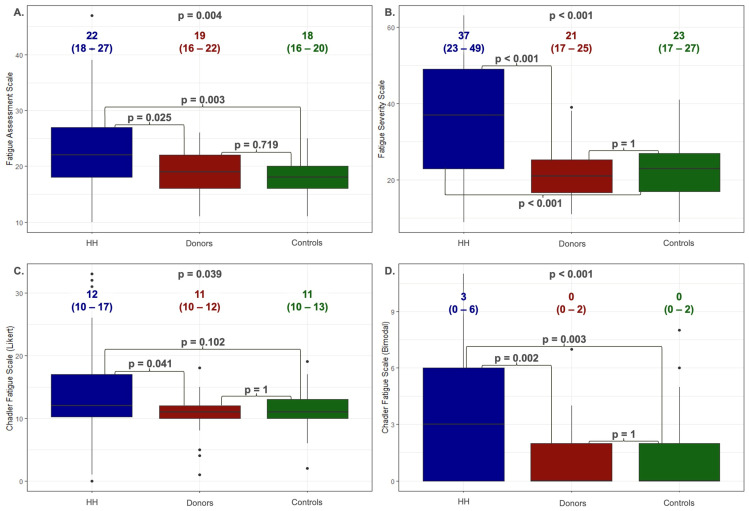
The values obtained on fatigue scales by patients with HH, donors, and controls (the Kruskal–Wallis test with Dunn’s post hoc test). Data are presented as the medians (25th–75th percentile). HH—hereditary hemochromatosis patients; controls—control group; donors—blood donors. (**A**)—Fatigue Assessment Scale; (**B**)—Fatigue Severity Scale; (**C**)—Chalder Fatigue Scale (Likert scoring method); (**D**)—Chalder Fatigue Scale (bimodal scoring method).

Figure 2 shows the values obtained using the Kruskal–Wallis test with Dunn’s post hoc test on the fatigue scales by the patients with HH, blood donors (donors), and healthy people (controls). The HH patients presented significantly higher scores on the FAS scale (22 (8–27)) compared to the donors (19 (16–22)) and controls (18 (16–20)). Those patients with HH also showed significantly higher parameters within the FSS scale (HH: 37 (23–49) vs. donors: 21 (17–25) vs. controls: 23 (17–27)) and CFQ in both the Likert and bimodal scores (Likert: HH: 12 (10–17) versus donors: 11 (10–12) vs. controls: 11 (10–13); bimodal: HH: 3 (0–6) vs. donors: 0 (0–2) vs. controls: 0 (0–2)). No statistically significant differences were observed between the donors and controls.

Table 3 shows the values obtained on the fatigue scales by the patients divided by three types of mutation—homozygotes C282Y/C282Y, heterozygotes C282Y/H63D, and homozygotes H63D/H63D (the Kruskal–Wallis test). No statistically significant differences were observed between the groups.

**Figure 3:**
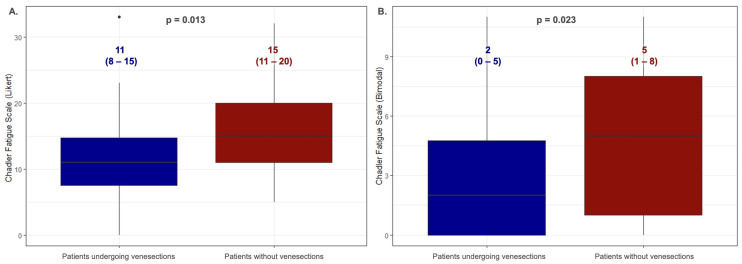
Comparison of the values obtained on fatigue scales by patients with HH divided by treatment with therapeutic venesections (the U Mann–Whitney test). Data are presented as the medians (25th–75th percentile). (**A**)—Chalder Fatigue Scale (Likert scoring method); (**B**)—Chalder Fatigue Scale (bimodal scoring method).

Figure 3 presents a comparison of the values obtained on the fatigue scales by the treated and untreated patients with HH (the U Mann–Whitney test). Those patients declaring current treatment with venesections had statistically significant lower fatigue than those not treated with this method only in the CFQ; for the Likert scoring method: 11 (8–15) vs. 15 (11–20); *p* = 0.013, and the bimodal scoring method: 2 (0–5) vs. 5 (1–8); *p* = 0.023. Sixty-nine percent of the people undergoing venesection therapy were patients with the C282Y/C282Y genotype. In the second group, the C282Y/C282Y genotype was present in 50% of the patients. Additionally, no statistically significant differences were observed in the iron and ferritin levels between the groups (*p* = 0.298 and *p* = 0.388, respectively). There were no statistically significant differences in the age of the compared groups: 39 (31–53) vs. 45 (39–58) (*p* = 0.180).

**Figure 4:**
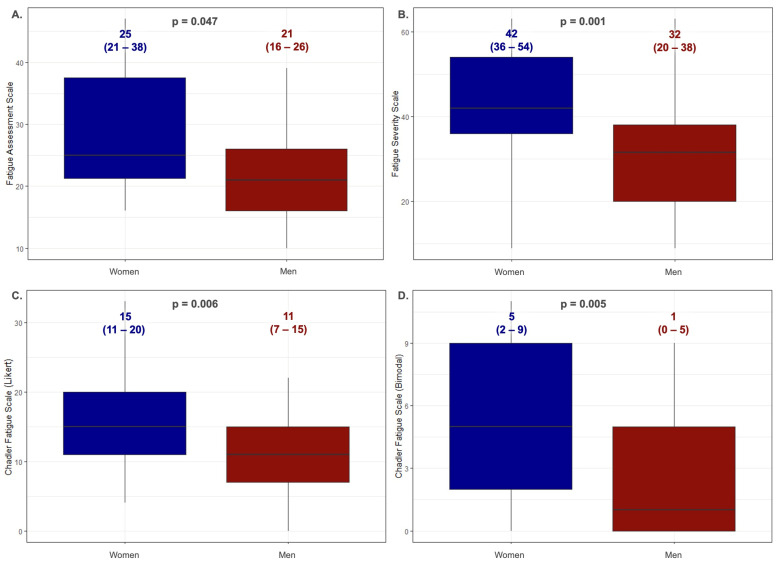
Comparison of the values obtained on fatigue scales by patients with HH divided by sex (the U Mann–Whitney test). Data are presented as the medians (25th–75th percentile). (**A**)—Fatigue Assessment Scale; (**B**)—Fatigue Severity Scale; (**C**)—Chalder Fatigue Scale (Likert scoring method); (**D**)—Chalder Fatigue Scale (bimodal scoring method).

Figure 4 shows the values obtained on the fatigue scales by the patients with HH divided by their sex (the U Mann–Whitney test). The women presented significantly higher scores compared to the men in each of the scales: for FAS 25 (21–38) vs. 21 (16–26), *p* = 0.047; for FSS: 42 (36–54) vs. 32 (20–38) *p* = 0.001; for CFQ (Likert): 15 (11–20) vs. 11 (7–15), *p* = 0.006; for CFQ (bimodal): 5 (2–9) vs. 11 1 (0–5), *p* = 0.005. The age of the women was higher than the men: 48 (38–61) vs. 41 (31–51); *p* = 0.001.

Table 4 shows the values obtained on the fatigue scales by the patients divided according to their age. No statistically significant differences were observed between the age groups. The age ranges presented are derived from a survey question included in the general part of the patient survey, categorized into frequently used groups.

### 3.2. Reliability Analysis and Validation of the Fatigue Assessment Scales Were Implemented

The Cronbach alpha reliability coefficient for the HH patients shows high degrees of internal consistency on each of the scales (for FAS: 0.923; for FSS: 0.95; for CFQ: 0.927). When deleting one item of the FSS, the Cronbach alpha values did not change markedly (range 0.908–0.927 for FSS; range 0.939–0.954 for FSS; range 0.916–0.925 for CFQ; Tables S1–S3).

A factor analysis conducted solely on the patient data revealed the unidimensional structure of the questionnaires used in the study. Applying Kaiser’s criterion, which excludes factors with an eigenvalue of less than 1, a single factor was derived, explaining the total variance between 58.2% and 71.7% depending on the scale analyzed (Tables S4–S6). The correlations between the scales are shown in Table S7. The construct validity was supported by high Spearman’s rank correlation coefficients across all the scales.

## 4. Discussion

The main finding of our study is that the patients with HH present significantly greater fatigue than the healthy controls and blood donors, which was confirmed by the points obtained on each of the scales used in the study with the excellent internal consistency and reliability of each of the implemented scales. According to our current knowledge, this is the first research aiming to create a fatigue profile in patients with HH using the FAS, FSS, and CFQ scales.

The majority of the HH patients (71%) reported fatigue. These patients consistently scored significantly worse on each scale than those patients who did not report this symptom. The HH patients indicated significantly worse fatigue parameters than the control group (Figure 1). The fatigue severity did not differ based on the type of HFE gene mutation. Examining the patients with the most common mutations (C282Y/C282Y, C282Y/H63D, and H63D/H63D), we found no differences in fatigue severity across these groups (Table 3). This result is astonishing because C282Y/C282Y homozygotes usually present the most severe forms of the disease, which are associated with higher iron and ferritin values [25]. The significance of this comparison is supported by our findings that there is no relationship between the level of iron metabolism parameters and the severity of fatigue.

Similarly, our previous studies did not demonstrate a relationship between iron metabolism parameters and echocardiographic indices [26]. The lack of a relationship between the iron levels and degree of fatigue indicates that HH is a complex process of iron overload, not a simple correlation between the iron metabolism parameters and the symptoms presented by patients. When comparing patients treated with venesections to those who did not undergo such therapy, a significant difference was observed only in the CFQ (Figure 3). The results indicated more significant fatigue among the untreated patients. This finding is logical as the disease in untreated patients is more advanced, leading to more organ complications and, consequently, higher levels of fatigue.

A significant finding of our study is that women with HH experienced more significant fatigue than men (Figure 4). That result is surprising considering that menstruation in women typically delays iron accumulation, which suggests lower fatigue levels until menopause, hysterectomy, or long-term use of continuous oral contraception. However, our results might be explained by the higher average age of the women in our study. Despite this, women tend to present less severe disease manifestations and are diagnosed later compared to men. The long-term observation of a large cohort of individuals with a homozygous C282Y HFE gene mutation for over 12 years revealed iron overload in 28.4% of the men but just 1.2% of the women [27]. On the other hand, the higher fatigue levels in the surveyed women suggest that factors beyond iron metabolism parameters may influence the severity of fatigue in these patients. This is consistent with our results showing no correlation between fatigue and iron and ferritin concentrations, indicating that HH symptoms arise in a complex, multifactorial manner, not solely limited to disturbances in iron management parameters.

Some other significant insights result from the analysis of patients across specific age groups. It was found that there is no statistically significant difference in the scores obtained on the fatigue assessment scales among patients of different ages (Table 4). The obtained results further emphasize the importance of comparing patients by gender, reducing the significance of the higher age among the women included in the analysis. The lack of age-related exacerbation of fatigue in our study group contrasts with the general population, where fatigue severity typically increases with age due to higher rates of chronic diseases, reduced endurance, and increased frailty [28]. Additionally, we did not observe a strong correlation between the year from diagnosis and fatigue severity. However, the treated patients showed less fatigue on the CFQ than the untreated patients, indicating that, regardless of disease duration or diagnosis timing, individuals with HH, especially those untreated, tend to experience a higher level of fatigue than the general population.

The donation patterns of blood donors resemble those of venesection therapy. Therefore, we chose to compare HH patients with the control group and blood donors. The HH patients exhibited significantly higher fatigue levels than the blood donors (Figure 2). It is worth noting that, unlike the control group, which was matched to the study based on age and gender, the blood donors were notably younger. Interestingly, we did not observe any differences in fatigue severity between the controls and donors across any of the scales used.

All the scales used in the presented study were characterized by excellent internal consistency and reliability, as confirmed by the high Cronbach’s alpha values: 0.923 for the FAS, 0.95 for the FSS, and 0.927 for the CFQ (Tables S1–S3). Our results are consistent with the FAS, FSS, and CFQ characteristics presented in the literature assessing fatigue severity, including patients with sarcoidosis and multiple sclerosis [2,3,9,14].

The construct validity measures of the FAS, FSS, and CFQ were confirmed by moderate Spearman’s rank coefficient correlations between each scale and BMI, age, years from diagnosis, ferritin, and the iron concentrations (Table 2 and Table S7). The factorial validity of the scales within the patient group confirmed a unidimensional structure among the HH patients, consistent with the previous findings in other clinical populations [2,3,9,14]. The scales’ convergent validity appeared to be exquisite. Those patients who reported feelings of fatigue were objectively more tired in the scales implemented in the study. Among the scales compared, the FSS exhibited the highest reliability and internal consistency coefficients. This suggests that it may be the most appropriate questionnaire for assessing fatigue in the studied patient group. However, the specific cause-and-effect relationship requires examination in the future.

The CFQ scale showed significant differences depending on the scoring method. While the Likert method revealed no significant differences between the HH patients and the control group, the bimodal method did show a statistically significant difference. The bimodal scoring method, which may better capture fatigue severity in HH patients, assigns values of 0 or 1 based on symptom severity, potentially offering a more accurate assessment for this population, showing how many items included in the scale occur in a particular case.

## 5. Clinical Implications

Fatigue is the primary symptom in modern patients with HH. This underscores the importance of having a reliable tool to assess this symptom. Currently, no literature data are available regarding the objective measurement of fatigue in this patient group. The FAS, FSS, and CFQ appeared to have good validity and reliability in HH. The results indicate that the scale used in the study may be a simple and reliable instrument for assessing and quantifying fatigue for clinical and research purposes in HH patients. Regardless of gender, age, or type of mutation, each patient should undergo a thorough assessment, including an evaluation of fatigue levels. Accurate assessment of fatigue in patients is crucial for improving their diagnosis, planning their treatment, and monitoring their health outcomes, leading to personalized care and enhanced quality of life. The results indicate that the fatigue mechanism in patients with HH is a multifactorial problem and requires further research, which was the first implication of the fatigue assessment scales in the HH population, thus confirming their excellent internal consistency and reliability.

## 6. Study Limitations

Our study limitation is the relatively low sample size resulting from the highly selective enrollment process. Therefore, we could not perform some analyses, for example, for the C282Y/WT mutation. Additionally, we did not measure the iron metabolism parameters in the control group or blood donors. This omission resulted from the inclusion criteria limiting the participants to healthy individuals. Moreover, blood donors undergo rigorous selection processes, reducing the likelihood of encountering individuals with abnormal results. The next limitation of our study is that we did not measure the HbA1c and testosterone levels in our patients, nor did we have information about their alcohol consumption. Another limitation concerns the study’s design as we collected data from various individuals at a single point in time, making it impossible to detect changes over time. This also led to the analysis of patients at different stages of the disease and treatment. We plan to conduct a longitudinal analysis in the future.

## 7. Conclusions

Fatigue is a predominant symptom in patients with HH, significantly surpassing that observed in healthy individuals. Given the scarcity of data on quantifying fatigue in this population, this study emphasizes the lack of variations in fatigue severity based on genotype or age. However, women report higher degrees of fatigue than men. The FAS, FSS, and CFQ scales presented excellent validity and reliability, making them valuable tools for assessing fatigue in HH patients.

## Figures and Tables

**Table 1:** The HH patients’ characteristics at the time of first contact.

	HH All n = 79
Age	44 (34–55)
Male sex [%]	53
Patients reporting fatigue [%]	71
Hypertension [%]	22
Liver cirrhosis [%]	18
Active treatment with venesections [%]	56
Iron [ug/dL]	171 (120–208)
Ferritin [ng/mL]	151 (72–358)
Haemoglobin [mg/dL]	15.3 (14.3–16)
ASPAT [U/L]	27 (20–49)
ALAT [U/L]	32 (19–51)

Data are presented as the medians (25th–75th percentile). ALAT—alanine aminotransferase; ASPAT—aspartate aminotransferase.

**Table 2:** Correlations between each scale and BMI, age, ferritin, and iron levels.

	Fatigue Assessment Scale	Fatigue Severity Scale	Chadler Fatigue Scale (Likert)	Chadler Fatigue Scale (Bimodal)
BMI	r = −0.055 (*p* = 0.598)	r = 0.075 (*p* = 0.405)	r = 0.002 (*p* = 0.979)	r = −0.042 (*p* = 0.645)
Age	r = −0.151 (*p* = 0.140)	r = 0.122 (*p* = 0.164)	r = 0.042 (*p* = 0.635)	r = 0.061 (*p* = 0.489)
Iron ug/dL	r = −0.168 (*p* = 0.344)	r = −0.102 (*p* = 0.411)	r = −0.016 (*p* = 0.901)	r = −0.033 (*p* = 0.797)
Ferritin ng/mL	r = 0.321 (*p* = 0.073)	r = 0.274 (*p* = 0.030)	r = 0.135 (*p* = 0.303)	r = 0.180 (*p* = 0.170)
Year from diagnosis	r = 0.109 (*p* = 0.478)	r = 0.204 (*p* = 0.075)	r = 0.312 (*p* = 0.006)	r = 0.250 (*p* = 0.033)

r—the Spearman rank correlation coefficient; *p*—probability value.

**Table 3:** Comparison of the values obtained on fatigue scales by patients with HH divided by type of mutation.

Scale	C282Y/C282Y n = 49	C282Y/H63D n = 16	H63D/H63D n = 12	*p*-Value
Fatigue Assessment Scale	21 (18–29)	21 (20–26)	24 (23–27)	0.666
Fatigue Severity Scale	38 (26–51)	28 (17–47)	36 (29–44)	0.4336
Chadler Fatigue Scale (Likert)	12 (11–19)	11 (19–15)	15 (11–16)	0.5587
Chadler Fatigue Scale (Bimodal)	3 (1–7)	1 (0–5)	4 (1–6)	0.4241

The Kruskal–Wallis test data are presented as the medians (25th–75th percentile).

**Table 4:** Comparisons of the values obtained on fatigue scales by patients with HH according to their age.

Scale	Age 18–25 n = 6	Age 26–45 n = 38	Age 46–65 n = 12	Age >65 n = 6	*p*-Value
Fatigue Assessment Scale	21 (21–24)	25.5 (20–37)	21 (16–25)	21 (16–22)	0.2016
Fatigue Severity Scale	35 (31–46)	37 (23–51)	36 (26–46)	37 (36–53)	0.8914
Chadler Fatigue Scale (Likert)	15 (12–19)	12 (8–17)	14 (11–18)	11 (6–14)	0.4013
Chadler Fatigue Scale (Bimodal)	4 (2–7)	2 (0–6)	3 (0–6)	2 (1–5)	0.949

The Kruskal–Wallis test data are presented as the medians (25th–75th percentile).

## Data Availability

Data are available from the corresponding author upon reasonable request.

## Supplementary Materials

The following supporting information can be downloaded at https://www.mdpi.com/article/10.3390/jcm13185544/s1, Figure S1: A translation-backtranslation procedure of the polish version of Fatigue Assessment Scale; Figure S2: A translation-backtranslation procedure of the polish version of Chalder Fatigue Scale; Figure S3: translation-backtranslation procedure of the polish version of Fatigue Severity Scale; Table S1: Cronbach’s alpha for Fatigue Assessment Scale (the entire scale, and when deleting one item of the scale); Table S2: Cronbach’s alpha for Fatigue Severity Scale (the entire scale, and when deleting one item of the scale); Table S3: Cronbach’s alpha for Chalder Fatigue Scale (the entire scale, and when deleting one item of the scale); Table S4: Principal component analyses with varimax rotation for FAS.; Table S5: Principal component analyses with varimax rotation for FSS.; Table S6: Principal component analyses with varimax rotation for CFQ; Table S7: Correlations between results obtained in each scale.
