# Supplementary material for: Fatigue Assessment in Patients with Hereditary Hemochromatosis: First Use of the Popular Diagnostic Tools
Source: J Clin Med. 2024 Sep 19;13(18):5544. doi: 10.3390/jcm13185544 (PMC11432497; doi:10.3390/jcm13185544)
Supplement: Supplementary file 1 [file jcm-13-05544-s001.zip › jcm-3186459-supplementary.pdf]

Figure S1. A translation-backtranslation procedure of the polish version of Fatigue Assessment Scale.

Fatigue Assessment Scale (FAS)					
QUESTION	NEVER	SOMETIMES	REGULARY	OFTEN	ALWAYS
I am bothered by fatigue.					
I get tired very quickly.					
I don't do much during the day.					
I have enough energy for everyday life.					
Physically, I feel exhausted.					
I have problems to start things.					
I have problems to think clearly.					
I feel no desire to do anything.					
Mentally, I feel exhausted.					
When I am doing something, I can concentrate quite well.					

translation-backtranslation procedure

Fatigue Assessment Scale (FAS)					
PYTANIE	NIGDY	CZASAMI	REGULARNIE	CZĘSTO	ZAWSZE
Cierpię z powodu zmęczenia					
Męczę się bardzo szybko					
Jestem mało aktywny(-na) w ciągu dnia					
Mam wystarczająco energii do codziennego życia					
Fizycznie czuję się wyczerpany(-na)					
Mam problem z rozpoczęciem czynności					
Mam problemy z jasnym myśleniem					
Brakuje mi chęci do zrobienia czegokolwiek					
Psychicznie jestem wyczerpany(-na)					
W trakcie wykonywania czynności koncentruję się całkiem dobrze					

Figure S2. A translation-backtranslation procedure of the polish version of Chalder Fatigue Scale.

Chadler Fatigue Scale (CFS)				
QUESTION	Less than usual	No more than usual	More than usual	Much more than usual
Do you have problems with tiredness?				
Do you need to rest more?				
Do you feel sleepy or drowsy?				
Do you have problems starting things?				
Do you lack energy?				
Do you have less strength in your muscles?				
Do you feel weak?				
Do you have difficulties concentrating?				
Do you make slips of the tongue when speaking?				
Do you find it more difficult to find the right word?				
	Less than usual	No more than usual	More than usual	Much more than usual
How is your memory?				

translation-backtranslation procedure

Chadler Fatigue Scale (CFS)				
PYTANIE	MNIEJ NIŻ ZWYKLE	TAK JAK ZWYKLE	BARDZIEJ NIŻ ZWYKLE	DUŻO BARDZIEJ NIŻ ZWYKLE
Czy ma Pan/Pani problemy ze zmęczeniem?				
Czy potrzebuje Pan/Pani więcej odpoczynku?				
Czy czuje się Pan/Pani senny(-a) lub ospały(-a)?				
Czy ma Pan/Pani problemy z rozpoczęciem czynności?				
Czy odczuwa Pan/Pani brak energii?				
Czy odczuwa Pan/Pani osłabienie siły mięśniowej?				
Czy czuje się Pan/Pani słaby(-a)?				
Czy ma Pan/Pani trudności z koncentracją?				
Czy przejęzycza się Pan/Pani w trakcie rozmowy?				
Czy trudniej jest Panu/Pani znaleźć właściwe słowo?				
	LEPIEJ NIŻ ZWYKLE	TAK JAK ZWYKLE	GORZEJ NIŻ ZWYKLE	DUŻO GORZEJ NIŻ ZWYKLE
Jak ocenia Pan/Pani swoją pamięć?				

Figure S3. A translation-backtranslation procedure of the polish version of Fatigue Severity Scale.

Fatigue Severity Scale (FSS)							
QUESTION	SIGNIFICATION OF SYMPTOMS						
My motivation is lower when I am fatigued.	1	2	3	4	5	6	7
Exercise brings on my fatigue.	1	2	3	4	5	6	7
I am easily fatigued.	1	2	3	4	5	6	7
Fatigue interferes with my physical functioning.	1	2	3	4	5	6	7
Fatigue causes frequent problems for me.	1	2	3	4	5	6	7
My fatigue prevents sustained physical functioning.	1	2	3	4	5	6	7
Fatigue interferes with carrying out certain duties and responsibilities.	1	2	3	4	5	6	7
Fatigue is among my three most disabling symptoms.	1	2	3	4	5	6	7
Fatigue interferes with my work, family, or social life.	1	2	3	4	5	6	7

translation-backtranslation procedure

Fatigue Severity Scale (FSS)							
PYTANIE	SKALA NASILENIA OBJAWÓW						
Moja motywacja jest niższa, kiedy jestem zmęczony	1	2	3	4	5	6	7
Ćwiczenia wywołują moje zmęczenie	1	2	3	4	5	6	7
Łatwo się męczę	1	2	3	4	5	6	7
Zmęczenie przeszkadza w mojej aktywności fizycznej	1	2	3	4	5	6	7
Zmęczenie jest dla mnie częstym problemem	1	2	3	4	5	6	7
Zmęczenie uniemożliwia mi wykonywanie długotrwałego wysiłku fizycznego	1	2	3	4	5	6	7
Zmęczenie zakłóca wykonywanie przeze mnie niektórych obowiązków	1	2	3	4	5	6	7
Zmęczenie jest jednym spośród trzech dolegliwości najbardziej ograniczających Pana/Pani funkcjonowanie	1	2	3	4	5	6	7
Zmęczenie przeszkadza w mojej pracy, życiu rodzinnym lub towarzyskim	1	2	3	4	5	6	7

Table. S1. Cronbach's alpha for Fatigue Assessment Scale (the entire scale, and when deleting one item of the scale).

Cronbach's alpha	0.923
Fatigue Assessment Scale Q1	0.908 (0.829-0.946)
Fatigue Assessment Scale Q2	0.916 (0.848-0.951)
Fatigue Assessment Scale Q3	0.927 (0.862-0.957)
Fatigue Assessment Scale Q4	0.918 (0.846-0.951)
Fatigue Assessment Scale Q5	0.914 (0.838-0.948)
Fatigue Assessment Scale Q6	0.912 (0.83-0.946)
Fatigue Assessment Scale Q7	0.912 (0.833-0.949)
Fatigue Assessment Scale Q8	0.911 (0.821-0.947)
Fatigue Assessment Scale Q9	0.911 (0.832-0.946)
Fatigue Assessment Scale Q10	0.922 (0.858-0.952)

Q1-10 item deleted.

Table S2. Cronbach's alpha for Fatigue Severity Scale (the entire scale, and when deleting one item of the scale).

Cronbach's alpha	0.946
Fatigue Severity Scale Q1	0.947 (0.924-0.963)
Fatigue Severity Scale Q2	0.954 (0.934-0.97)
Fatigue Severity Scale Q3	0.943 (0.916-0.961)
Fatigue Severity Scale Q4	0.939 (0.915-0.957)
Fatigue Severity Scale Q5	0.939 (0.911-0.958)
Fatigue Severity Scale Q6	0.942 (0.918-0.96)
Fatigue Severity Scale Q7	0.943 (0.916-0.961)
Fatigue Severity Scale Q8	0.942 (0.918-0.958)
Fatigue Severity Scale Q9	0.944 (0.917-0.961)

Q1-9 item deleted.

Table. S3. Cronbach's alpha for Chalder Fatigue Scale (the entire scale, and when deleting one item of the scale).

Cronbach's alpha	0.927
Chadler Fatigue Scale Q1	0.916 (0.863-0.948)
Chadler Fatigue Scale Q2	0.919 (0.868-0.947)
Chadler Fatigue Scale Q3	0.921 (0.87-0.948)
Chadler Fatigue Scale Q4	0.92 (0.872-0.949)
Chadler Fatigue Scale Q5	0.916 (0.864-0.944)
Chadler Fatigue Scale Q6	0.922 (0.87-0.95)
Chadler Fatigue Scale Q7	0.917 (0.863-0.947)
Chadler Fatigue Scale Q8	0.921 (0.871-0.947)
Chadler Fatigue Scale Q9	0.922 (0.873-0.949)
Chadler Fatigue Scale Q10	0.92 (0.868-0.948)
Chadler Fatigue Scale Q11	0.925 (0.874-0.953)

Q1-11 item deleted.

Table S4. Principal component analyses with varimax rotation for FAS.

	loadings
**Fatigue Assessment Scale Q1**	0.887
**Fatigue Assessment Scale Q2**	0.756
**Fatigue Assessment Scale Q3**	0.562
**Fatigue Assessment Scale Q4**	0.717
**Fatigue Assessment Scale Q5**	0.794
**Fatigue Assessment Scale Q6**	0.835
**Fatigue Assessment Scale Q7**	0.817
**Fatigue Assessment Scale Q8**	0.860
**Fatigue Assessment Scale Q9**	0.851
**Fatigue Assessment Scale Q10**	0.655
**Variance [%]**	60.8

Q1-10 item deleted.

Table S5. Principal component analyses with varimax rotation for FSS.

	loadings
**Fatigue Severity Scale Q1**	0.791
**Fatigue Severity Scale Q2**	0.649
**Fatigue Severity Scale Q3**	0.861
**Fatigue Severity Scale Q4**	0.911
**Fatigue Severity Scale Q5**	0.912
**Fatigue Severity Scale Q6**	0.867
**Fatigue Severity Scale Q7**	0.867
**Fatigue Severity Scale Q8**	0.879
**Fatigue Severity Scale Q9**	0.851
**Variance [%]**	71.7

Q1-9 item deleted.

Table S6. Principal component analyses with varimax rotation for CFQ.

	loadings
**Chadler Fatigue Scale Q1**	0.839
**Chadler Fatigue Scale Q2**	0.782
**Chadler Fatigue Scale Q3**	0.739
**Chadler Fatigue Scale Q4**	0.762
**Chadler Fatigue Scale Q5**	0.855
**Chadler Fatigue Scale Q6**	0.728
**Chadler Fatigue Scale Q7**	0.814
**Chadler Fatigue Scale Q8**	0.749
**Chadler Fatigue Scale Q9**	0.711
**Chadler Fatigue Scale Q10**	0.751
**Chadler Fatigue Scale Q11**	0.637
**Variance [%]**	58.2

Q1-11 item deleted.

Table S7. Correlations between results obtained in each scale.

	Fatigue Assessment Scale	Fatigue Severity Scale	Chadler Fatigue Scale (Linkert)	Chadler Fatigue Scale (Bimodal)
**Fatigue Assessment Scale**	r = 1.000<br>p <0.001	r = 0.846<br>p <0.001	r = 0.656<br>p <0.001	r = 0.656<br>p <0.001
**Fatigue Severity Scale**	r = 0.846<br>p <0.001	r = 1.000<br>p <0.001	r = 0.587<br>p <0.001	r = 0.598<br>p <0.001
**Chadler Fatigue Scale (Linkert)**	r = 0.656<br>p <0.001	r = 0.587<br>p <0.001	1.000<br>p <0.001	r = 0.708<br>p <0.001
**Chadler Fatigue Scale (Bimodal)**	r = 0.656<br>p <0.001	r = 0.598<br>p <0.001	r = 0.708<br>p <0.001	r = 1.000<br>p <0.001

r - the Spearman rank correlation coefficient; p - probability value
